# Home-based rehabilitation after hip or knee arthroplasty among rural older adults in China: a qualitative study

**DOI:** 10.3389/fpubh.2026.1957794

**Published:** 2026-10-19

**Authors:** Yanchi Wang, Wei Huang, Jianhua Deng, Jian Gu

**Affiliations:** 1 Affiliated Nantong Hospital of Shanghai University (The Sixth People’s Hospital of Nantong), Nantong, Jiangsu, China; 2 Medical School of Nantong University, Nantong University, Nantong, Jiangsu, China

**Keywords:** continuity of care, hip replacement, home-based rehabilitation, knee replacement, qualitative study, rural older adults

## Abstract

**Background:**

Older adults in rural China may face difficulties accessing continuous and professional rehabilitation after hip or knee replacement. This study explored their experiences of home-based rehabilitation, focusing on barriers, coping strategies, and healthcare support gaps.

**Method:**

A descriptive phenomenological study was conducted from January to March 2025 in the orthopaedic outpatient clinics of two tertiary hospitals in Nantong, China. Twenty-one rural adults aged 60 years or older who had undergone primary hip or knee replacement within the previous 6 months were recruited using maximum variation sampling. Face-to-face semi-structured interviews were conducted, and data were analysed using Colaizzi’s seven-step method.

**Results:**

Three themes and nine subthemes were identified: (1) Challenges in Physical Rehabilitation: Pain, Functional Limitations, and Exercise Adaptations, including The Helplessness and Grassroots Wisdom in Pain Management, Adaptations and Misunderstandings of Prescribed Exercises, and Loss of Daily Function and Role Changes; (2) Limited Family and Community Support, including Emotional Debt in Familial Care, Limited Rehabilitation Capacity in Village Clinics, and Reluctance to Seek Neighbor Support; and (3) Negotiating Traditional Beliefs and Modern Rehabilitation, including Barriers to Formal Rehabilitation and Reliance on Traditional Remedies, Views on Aging and Prioritizing Family Needs, and Folk Beliefs and Medical Advice. These findings showed that participants’ rehabilitation experiences were shaped by physical limitations, discontinuity of family and community support, and the interaction between traditional beliefs and modern rehabilitation practices.

**Conclusion:**

Home-based rehabilitation after hip or knee replacement among rural older adults is shaped by physical, family, cultural, and service-related factors. Potential approaches include accessible rehabilitation materials, structured follow-up, caregiver support, and stronger coordination between tertiary hospitals and rural primary care services; however, their feasibility and effectiveness require further evaluation.

## 1 Introduction

The aging of the population is advancing rapidly, and it is estimated that, by 2050, one in every five people globally will be aged 60 or above (1, 2). This aging trend is particularly pronounced in China, where the older adults population is growing at a rapid pace (3). Osteoarthritis (OA) is the third most disabling musculoskeletal disease globally, causing 17.1 million years of disability in 2010 (4). The knee joint is the most frequently affected (5), followed by the hip and hand joints (5). Hip and knee OA are not only painful, progressive, degenerative joint diseases but also the most common causes of ambulatory functional impairment (6–8). This age-dependent progression is attributed to cumulative mechanical stress, chondrocyte senescence, and age-related declines in synovial fluid viscosity, which collectively accelerate cartilage degradation (9–12). Total hip or knee arthroplasty (THA/TKA) is a common surgical intervention for advanced hip/knee OA. These surgeries generally can relieve pain and correct joint deformities (13), but they may also increase the risk of falls in older adults (13, 14). Due to fear of falling, compounded by comorbidities and muscle weakness caused by sarcopenia, older patients often experience delayed functional recovery (15, 16). This ultimately leads to a decline in quality of life and loss of independence. Given these challenges, postoperative rehabilitation is crucial for older adults to regain functionality and improve their overall wellbeing.

In rural areas of China, the challenges of postoperative rehabilitation are compounded by significant structural and individual vulnerabilities. Rural regions often suffer from limited medical resources, with fewer specialized rehabilitation facilities and healthcare professionals compared to urban areas (17). The geographical remoteness of these areas results in poor accessibility to healthcare services, further exacerbated by inadequate transportation infrastructure (18). Economic constraints also play a crucial role, as many rural families struggle to afford the costs associated with rehabilitation therapies and follow-up care (19). Moreover, the dissemination of rehabilitation knowledge is hindered by low levels of education and health literacy among the rural population (20, 21). On an individual level, older adults patients in rural areas often have lower educational attainment, which can lead to a lack of understanding of the importance of postoperative rehabilitation and adherence to prescribed care plans. Additionally, the traditional family structure in rural China, characterized by younger family members migrating to urban areas for work, leaves many older adults individuals with limited family support. This lack of support, combined with the psychological stress of dealing with chronic pain and disability, can significantly impair the recovery process.

Joint replacement surgery is typically followed by rehabilitation, which includes physical therapy, pedal-based programs, and continuous passive motion (22–24). However, there is still considerable debate over the most appropriate forms of rehabilitation. Loss of lower limb strength and function often occurs within the first month after joint replacement surgery, making early exercise crucial for functional recovery (25). While previous studies have reported that rehabilitation after joint replacement surgery is not associated with the final outcome (26), recent research has found that progressive resistance training and functional strengthening exercises can improve joint function while ensuring safety (27, 28). Therefore, adequate functional exercise can stimulate muscle growth, increase muscle strength, and promote the recovery of joint function (29–31). During this critical period, patients require intensive rehabilitation to regain strength, mobility, and independence. The role of family and community support in this recovery process cannot be overstated (32, 33). However, rural older adults patients face unique challenges that significantly impact their ability to engage in effective rehabilitation (34). These challenges include limited access to specialized rehabilitation facilities, economic constraints, and a lack of family support due to the migration of younger family members to urban areas for work. Additionally, rural areas often suffer from inadequate transportation infrastructure, making it difficult for older adults patients to travel to healthcare facilities for follow-up care and rehabilitation sessions (35). These structural and individual vulnerabilities compound the difficulties faced by rural older adults patients, often resulting in delayed or incomplete recovery. Despite the importance of rehabilitation and support systems, many rural older adults patients struggle to achieve satisfactory recovery outcomes (36). A large proportion of patients do not return to normal functional levels after surgery, which not only affects the quality of life of the patients but also places a considerable burden on their families and communities (37). However, most evidence on post-arthroplasty rehabilitation comes from urban or facility-based settings. Little is known about how rural older adults in China manage rehabilitation at home, what barriers they face, and what healthcare support is missing.

Given the multifaceted nature of the challenges faced by rural older adults patients undergoing joint replacement surgery, qualitative research offers a valuable approach to uncovering the complexities of their postoperative experiences. Unlike quantitative methods that focus on measurable outcomes, qualitative research allows for an in-depth exploration of the subjective experiences, perceptions, and coping strategies of individuals (38). This approach can provide rich insights into the lived realities of rural older adults patients, revealing the barriers and facilitators of effective rehabilitation (39). By employing qualitative methods such as in-depth interviews and participant observation, researchers can gain a nuanced understanding of the interplay between structural, cultural, and individual factors that shape the recovery process. Home-based rehabilitation serves as a critical component in the continuum of care for OA patients following joint replacement, particularly in resource-limited settings. It enables continuous functional training in a patient’s familiar environment, which is essential for consolidating surgical outcomes, preventing functional decline, and reducing readmission risks, especially when access to facility-based therapy is constrained. To address this gap, this study explores the shared experiences of home-based rehabilitation after hip or knee arthroplasty among rural older adults in China, rather than procedure-specific rehabilitation outcomes. Both THA and TKA require early functional exercise, pain management, and continuous support, and rural patients face similar structural barriers. The study focuses on the barriers they encounter, the strategies they use to cope, and the gaps in healthcare support. The research question was: How do rural older adults in China experience and manage home-based rehabilitation after hip or knee arthroplasty, and what physical, psychological, family, cultural, and health-system barriers and support gaps do they encounter? Psychological experience is treated as one component of this broader rehabilitation process.

## 2 Methods

### 2.1 Design

This was a descriptive phenomenological study using Colaizzi’s seven-step method. This approach was chosen to explore the lived experience of home-based rehabilitation among rural older adults after hip or knee arthroplasty. Participants’ experiences were understood within their social and cultural context, but the analytic procedure followed Colaizzi’s descriptive phenomenological method. Data were collected through semi-structured interviews to allow flexibility in exploring participants’ subjective perceptions while maintaining focus on core research questions.

### 2.2 Recruitment

This qualitative study was conducted in the orthopedic outpatient clinics of two tertiary hospitals located in Nantong City, Jiangsu Province. These hospitals function as regional medical centers and serve a substantial number of patients from surrounding rural areas, making them strategically appropriate for recruiting the target demographic of rural older adults patients. Participant recruitment and data collection took place face-to-face between January and March 2025 during scheduled postoperative follow-up visits. Upon prior discharge after their joint replacement surgery, all participants had received a standardized, written home-based rehabilitation plan from the orthopedic department. This plan provided foundational, joint-specific exercise guidance—such as ankle pumps, straight leg raises, and active range-of-motion exercises—commonly prescribed for recovery after total hip or total knee arthroplasty, which formed the basis for exploring their subsequent rehabilitation experiences at home. Inclusion criteria were: (1) age ≥ 60 years, (2) underwent primary total hip/knee arthroplasty within the past 6 months, (3) resided in rural areas (defined as non-urban administrative regions), (4) able to communicate verbally in Mandarin or local dialects. Exclusion criteria included: (1) cognitive impairment, (2) severe comorbidities limiting communication (e.g., advanced dementia, aphasia). A maximum variation sampling strategy was employed to ensure a diverse range of perspectives. Variation was sought with respect to age (60–69, 70–79, ≥80 years), sex, arthroplasty type (THA/TKA), postoperative month (1–3 months), marital status, living arrangement (alone, with spouse, with children), and education (primary school or below, junior high school or above). These characteristics were considered during recruitment to obtain a heterogeneous sample. A total of 21 participants were interviewed. Data saturation was reached after the 20th interview, when no new themes emerged. One additional participant was subsequently interviewed to confirm that no substantially new information or themes were identified. No repeat interviews were carried out. None of the approached patients refused participation or dropped out. Both THA and TKA patients were included because the study focused on shared experiences of home-based rehabilitation rather than procedure-specific outcomes. Joint-specific precautions and exercise prescriptions were explored during interviews.

### 2.3 Data collection

The interview guide, which is shown in Supplementary Material 1, was developed based on a review of the literature and consultation with experts in the field. It included open-ended questions about participants’ experiences and perceptions of home-based rehabilitation exercises, pain management, family and community support, as well as their experiences and views related to recovery. To enhance clarity, relevance, and appropriateness of the questions for the target population, the guide was pilot tested with two rural older adults patients who had undergone joint replacement surgery prior to the formal study. The pilot test confirmed that the questions were understandable and effectively elicited rich narratives about the rehabilitation experience. Minor adjustments were subsequently made to the wording and sequencing of some prompts based on this feedback. The interviews were conducted in a quiet consultation room after the participants’ outpatient visits, with each interview lasting approximately 45–60 min. Only the interviewer and the participant were present during the interviews. Data collection and analysis occurred concurrently in an iterative process, which allowed preliminary findings to inform subsequent interviews. Preliminary findings from early interviews informed slight refinements to the interview guide to better explore emerging themes in subsequent interviews. Data were captured through audio recordings, video recordings, and field notes. Audio recordings captured verbal content, video recordings captured non-verbal cues, and field notes recorded contextual observations and researchers’ reflections. These complementary sources were used together to enrich the data. During the interviews, researchers first confirmed the participants’ understanding of this prescribed plan before exploring their subsequent experiences, adherence, and any adaptations made during the home-based recovery phase. All interviews were audio- and video-recorded with consent, and transcripts were anonymized to ensure confidentiality.

### 2.4 Research team and reflexivity

The research team was multidisciplinary, comprising a nursing specialist with a Master of Nursing Science (YW, female), a clinical medicine student (WH, male), a university-based researcher with a Master of Clinical Medicine (JG, male), and a chief orthopedic surgeon (JD, male). The interviewers (YW and WH) had both received formal training in qualitative research methods. To mitigate potential influences of the researchers on data collection, post-interview debriefings were conducted to reflect on and bracket preconceptions. The emotional impact of the research on the interviewers was also acknowledged and managed through team discussions to ensure analytical rigor. Prior to the interviews, participants were informed about the researchers’ professional affiliations and the overarching goal of the study. However, the researchers did not disclose their specific personal hypotheses or individual research goals to the participants, so as not to inadvertently influence the participants’ narratives. We recognized that our professional backgrounds could influence the research. The clinical expertise of JD and JG might incline the analysis toward a biomedical perspective, while YW’s nursing background would foster a stronger focus on psychosocial aspects. To practice reflexivity and mitigate these potential biases, we maintained a reflective journal and held regular team discussions. This process allowed us to critically examine how our perspectives might shape data interpretation and ensure that the findings remained grounded in the participants’ experiences. No prior relationship existed between the interviewers and participants.

### 2.5 Data analysis

Within 24 h after each interview, two researchers transcribed the recordings and imported them into NVivo 12.2 for analysis. Transcripts were cross-checked against the original audio recordings to verify accuracy and data integrity. All data were anonymized during transcription, with identifying details removed or replaced by pseudonyms; encrypted storage and access controls were used throughout the study. Two researchers independently analyzed the data using Colaizzi’s seven-step method (40): (1) repeated reading of the transcripts to gain familiarity; (2) extraction of significant statements relevant to the research question; (3) formulation of meanings from these statements; (4) clustering of formulated meanings into theme clusters; (5) development of an exhaustive description of the phenomenon; (6) identification of the fundamental structure; and (7) participant validation. Differences in interpretation were discussed until consensus was reached; when necessary, a third researcher was consulted. Five participants were selected from the interviewed participants who were available for follow-up confirmation and represented both sexes and both THA and TKA groups. Because of potential literacy barriers among this rural older population, verbatim transcripts were not returned for review. Instead, the researchers orally summarized the preliminary findings and themes to these participants to confirm whether the interpretations accurately reflected their experiences. The final dataset included all 21 participant interviews. Interviews were conducted and analyzed in Chinese. Selected quotations were translated into English by two bilingual researchers and checked against the original transcripts. We also checked whether the main themes appeared in both the THA and TKA groups and noted procedure-specific differences where relevant. A formal subgroup analysis was not performed because of the small sample size and the qualitative design.

### 2.6 Ethical considerations

This study was approved by the Ethics Committee of the Sixth People’s Hospital of Nantong (Ethics Approval Number: NTLYLL2025007). The study adhered to the principles of the Declaration of Helsinki. Researchers explained the purpose and content of the study to the participants, who were assured of anonymity and confidentiality. Data were used solely for research purposes, and participants had the right to withdraw from the study at any time without penalty. All participants voluntarily participated in the study and provided written informed consent.

## 3 Results

A total of 21 participants were interviewed (female participants: *n* = 11, 52.4%; male participants: *n* = 10, 47.6%). The demographic characteristics of the participants are shown in Table 1 as follows. Themes were derived inductively from the data. Three core themes emerged: (1) Challenges in Physical Rehabilitation: Pain, Functional Limitations, and Exercise Adaptations; (2) Limited Family and Community Support; and (3) Negotiating Traditional Beliefs and Modern Rehabilitation. The coding tree structure developed from the data analysis is summarized in Figure 1.

**Table 1:** Demographic characteristics of the participants (*n* = 21).

Participants	Gender	Age	Surgical replacement types	Postoperative month	Marital status	Living arrangement	Education
P1	Female	78	Hip joint	1 month	Married	With spouse	No formal education
P2	Male	75	Knee joint	2 months	Divorced	Alone	Primary school
P3	Female	68	Knee joint	1 month	Married	With spouse	Primary school
P4	Male	82	Hip joint	1 month	Widowed	Alone	No formal education
P5	Female	80	Knee joint	1 month	Widowed	Alone	No formal education
P6	Male	74	Hip joint	1 month	Widowed	Alone	Primary school
P7	Male	77	Hip joint	3 months	Married	With spouse	Primary school
P8	Female	79	Hip joint	2 months	Married	With spouse	No formal education
P9	Female	85	Hip joint	2 months	Married	With spouse	No formal education
P10	Male	71	Hip joint	1 month	Married	With spouse	Primary school
P11	Male	70	Knee joint	2 months	Married	With spouse	Primary school
P12	Female	76	Knee joint	3 months	Married	With children	No formal education
P13	Male	81	Hip joint	1 month	Widowed	With children	No formal education
P14	Female	65	Knee joint	1 month	Divorced	Alone	Primary school
P15	Female	79	Knee joint	2 months	Married	With children	No formal education
P16	Female	73	Hip joint	3 months	Married	With spouse	Primary school
P17	Male	69	Knee joint	1 month	Married	With spouse	Primary school
P18	Female	65	Knee joint	2 months	Married	With spouse	Primary school
P19	Male	82	Hip joint	3 months	Widowed	With children	No formal education
P20	Male	82	Knee joint	3 months	Widowed	Alone	No formal education
P21	Female	87	Knee joint	2 months	Widowed	Alone	No formal education

**Figure 1:**
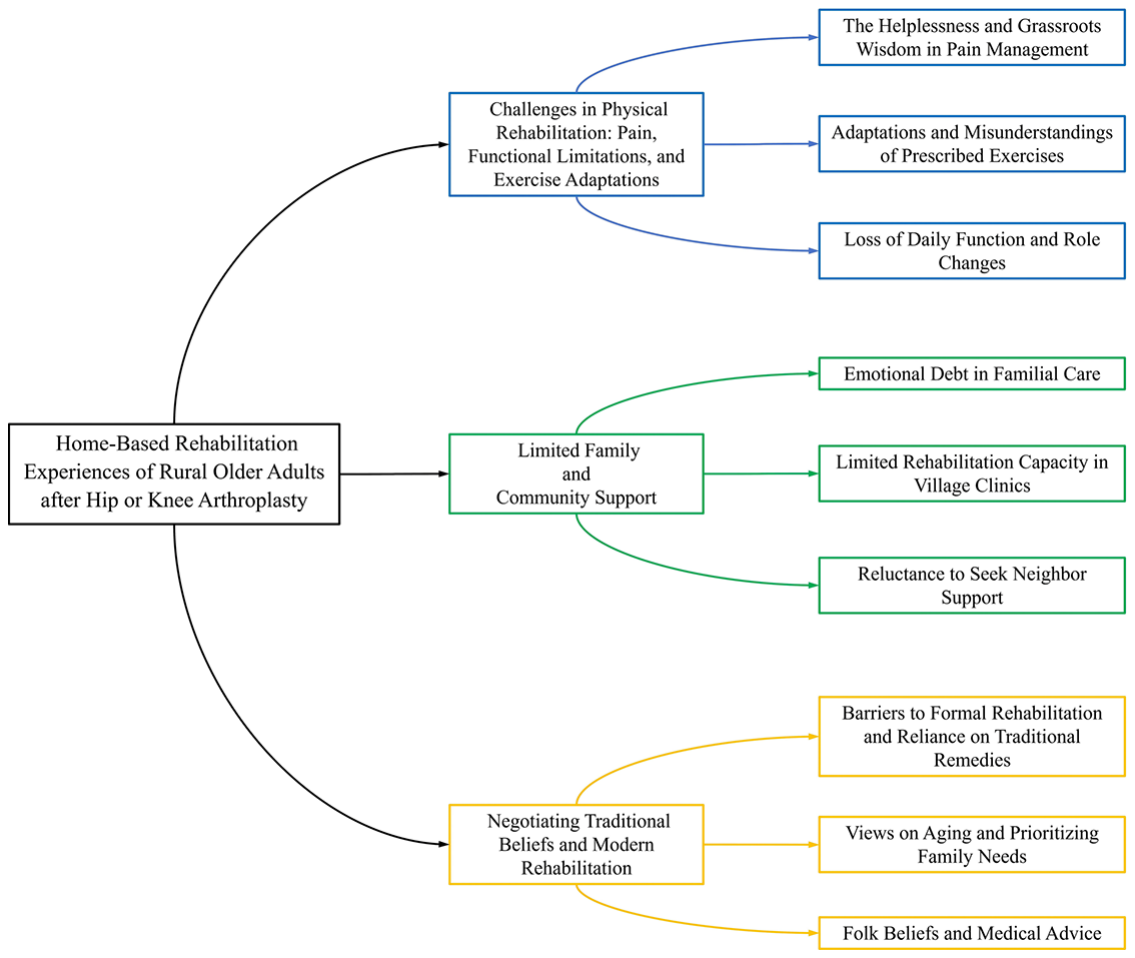
Thematic structure of home-based rehabilitation experiences among rural older adults after hip or knee arthroplasty.

### 3.1 Theme 1: Challenges in physical rehabilitation: pain, functional limitations, and exercise adaptations

Rural elders encountered significant barriers in their post-joint replacement recovery, driven by economic constraints, cultural perceptions of resilience, and insufficient rehabilitation resources. Their coping strategies often involved a blend of resourcefulness and resignation, which frequently exacerbated both physical and emotional strain.

#### 3.1.1 The helplessness and grassroots wisdom in pain management

Participants avoided formal pain management due to cost concerns and distrust in pharmaceuticals, relying instead on accessible traditional methods. Cultural narratives framing pain endurance as virtuous further normalized delayed recovery.

*“For pain that keeps me awake, I use a plaster. I have some leftover Yunnan Baiyao from last year, so I’ll just make do with that for now.”*

*(P3, female, 68 years, TKA, 1 month postoperatively)*

*“I use herbal poultices to reduce swelling and relieve pain. This method works slowly, but it doesn’t harm your organs like Western medicine does.”*

*(P6, male, 74 years, THA, 1 month postoperatively)*

*“The doctor gave me painkillers, but I only take them when the pain is unbearable. We old people have endured hardship all our lives, bearing a little pain is a virtue. If you can’t even handle this, you’ll be laughed at.”*

*(P7, male, 77 years, THA, 3 months postoperatively)*

#### 3.1.2 Adaptations and misunderstandings of prescribed exercises

Limited space and unclear guidance led participants to modify prescribed exercises, and some modifications deviated from the prescribed instructions.

*“The therapist said ‘leg lifts,’ but my bed is too high. I just swing my legs while sitting-it’s better than nothing.”*

*(P11, male, 70 years, TKA, 2 months postoperatively)*

*“I tried using a broomstick as a walker. It slipped, and I fell… now my son hides the broom.”*

*(P13, male, 81 years, THA, 1 month postoperatively)*

*“The doctor’s drawings were confusing. I thought ‘knee bends’ meant rocking in my chair.”*

*(P18, female, 65 years, TKA, 2 months postoperatively)*

#### 3.1.3 Loss of daily function and role changes

Participants reported difficulties with basic tasks and changes in daily roles, particularly reduced participation in previous household responsibilities.

*“I used to haul firewood. Now I can’t even carry my own chamber pot.”*

*(P4, male, 82 years, THA, 1 month postoperatively)*

*“At family meals, I used to serve everyone. Now I have to wait for my grandson to bring me the bowl. I feel like a guest in my own home.”*

*(P12, female, 76 years, TKA, 3 months postoperatively)*

*“My daughter-in-law thinks I take too long to bathe, so now I just wipe myself down every three or four days.”*

*(P15, female, 79 years, TKA, 2 months postoperatively)*

### 3.2 Theme 2: Limited family and community support

Support networks were fractured by migration, under-resourced rural healthcare, and cultural stigma around dependency. Elders struggled with guilt over “burdening” families while navigating superficial community aid.

#### 3.2.1 Emotional debt in familial care

Adult children’s urban migration left elders reliant on perfunctory check-ins, while spousal care often resulted in mutual physical decline.

*“My son works away and only comes home once a year. He says in the video call, ‘Mom, you need to exercise more, ‘but he does not realize how hard it is for me to even walk.?*

*(P1, female, 78 years, THA, 1 month postoperatively)*

*“My daughter sends money but never visits. Money can’t lift me off the floor when I fall.”*

*(P9, female, 85 years, THA, 2 months postoperatively)*

*“When my son calls, I always tell him I’m doing fine and that the recovery is going well. How can I tell him the truth? He has his own family and burdens to bear in the city. I don’t want to add to his worries.”*

*(P10, male, 71 years, THA, 1 month postoperatively)*

*“My spouse helped me walk and ended up falling, now he has a sore back, and I’m afraid to ask for his help again.”*

*(P16, female, 73 years, THA, 3 months postoperatively)*

#### 3.2.2 Limited rehabilitation capacity in village clinics

Village clinics had limited rehabilitation expertise and often provided general advice rather than targeted rehabilitation guidance. Participants reported a lack of specific rehabilitation support.

*“The nurse handed me a pamphlet in Mandarin-I can’t read. She said,‘Just follow the pictures.’”*

*(P5, female, 80 years, TKA, 1 month postoperatively)*

*“They check my blood pressure but never ask about my knee. What good is a strong heart if I can’t stand?”*

*(P12, female, 76 years, TKA, 3 months postoperatively)*

*“The village doctor told me, ‘Old bones heal slow.’ Is that science or an excuse?”*

*(P19, male, 82 years, THA, 3 months postoperatively)*

#### 3.2.3 Reluctance to seek neighbor support

Participants were reluctant to seek help from neighbours, often to avoid feeling indebted or appearing burdensome. Assistance, when received, tended to be short-term.

*“Neighbors helped me buy groceries twice, but I felt like I was in their debt, so later I preferred to just eat pickles.”*

*(P2, male, 75 years, TKA, 2 months postoperatively)*

*“I was afraid others would mock my son for being unfilial, so I didn’t dare to complain about the pain.”*

*(P8, female, 79 years, THA, 2 months postoperatively)*

*“Asking for help means admitting weakness. Better to suffer quietly.”*

*(P17, male, 69 years, TKA, 1 month postoperatively)*

### 3.3 Theme 3: Negotiating traditional beliefs and modern rehabilitation

Participants described tensions between traditional beliefs and modern medical advice. They often preferred familiar, low-cost remedies, and some deprioritized their own care in the context of aging and family financial priorities.

#### 3.3.1 Barriers to formal rehabilitation and reliance on traditional remedies

Geographic and financial barriers limited access to formal rehabilitation. Participants often relied on familiar and accessible traditional methods that provided immediate sensory feedback.

*“Western medicine suggested physiotherapy, but the town health center is too far away. Moxibustion is more convenient, and traditional methods from our ancestors are always reliable.”*

*(P7, male, 77 years, THA, 3 months postoperatively)*

*“The hot and spicy sensation from applying plasters makes me feel like it’s ‘working,’ which is more reassuring than cold, impersonal machines.”*

*(P20, male, 82 years, TKA, 3 months postoperatively)*

#### 3.3.2 Views on aging and prioritizing family needs

Participants sometimes attributed functional decline to aging and reduced their rehabilitation efforts. They also prioritized family financial needs over their own rehabilitation.

*“Why fix my hip? I’ll die soon anyway. Better save the money for my grandson’s textbooks.”*

*(P10, male, 71 years, THA, 1 month postoperatively)*

*“My son’s family is saving to buy a car. My rehabilitation is important, but their future is more important. I can manage with a limp; it’s not a big deal.”*

*(P19, male, 82 years, THA, 3 months postoperatively)*

*“I’m getting old, and spending too much money doesn’t make sense. As long as I can walk, that’s fine. I’m not thinking about rehabilitation.”*

*(P21, female, 87 years, TKA, 2 months postoperatively)*

#### 3.3.3 Folk beliefs and medical advice

Participants used folk beliefs to explain surgical outcomes and to gain a sense of control when medical information was limited.

*“The doctor can only fix the body, but the temple can fix the soul. If your soul is heavy, your body won’t heal properly. That’s why I go to pray for lightness.”*

*(P6, male, 74 years, THA, 1 month postoperatively)*

*“The monk at the temple said this is a karmic obstacle from my past life, and burning more incense will help.”*

*(P14, female, 65 years, TKA, 1 month postoperatively)*

*“The surgery date wasn’t chosen properly, it clashed with the Grand Duke Jupiter (a deity in Chinese folk astrology), that’s why the recovery is slow.”*

*(P21, female, 87 years, TKA, 2 months postoperatively)*

## 4 Discussion

This study identified three themes in the home-based rehabilitation experiences of rural older adults after hip or knee arthroplasty: physical challenges and exercise adaptations, limited family and community support, and negotiation between traditional beliefs and modern rehabilitation. These findings are consistent with existing evidence on rural aging, family migration, and primary care capacity in China.

Limited household resources and insufficient professional guidance appeared to contribute to participants’ adaptations of prescribed rehabilitation exercises, some of which deviated from the recommended instructions and may increase the risk of adverse outcomes (41). This finding is consistent with previous evidence suggesting that rehabilitation strategies need to account for patients’ living conditions and local resource constraints (42). Clearer and more accessible rehabilitation information, including illustrated instructions and simple verbal explanations, may help patients better understand prescribed exercises, particularly in settings where access to professional rehabilitation is limited (43–46). In addition, structured follow-up after discharge may provide opportunities to identify inappropriate exercise practices and address uncertainties arising during home-based rehabilitation. These approaches should be regarded as potential directions for service development, and their feasibility and effectiveness in rural settings require further evaluation.

The erosion of familial support associated with adult children’s migration reflects a broader challenge faced by many rural older adults. Although financial support may remain available, participants’ accounts showed that it could not substitute for physical assistance and emotional presence during rehabilitation, which is consistent with previous observations regarding limitations of remote family support (47). These findings suggest that home-based rehabilitation may be particularly vulnerable when family support is limited and local primary care has insufficient rehabilitation capacity. Family members may benefit from clearer guidance on how to provide basic and safe rehabilitation support at home, while rural patients may require more consistent access to professional advice after discharge. At the service level, closer communication and coordination between tertiary hospitals and rural primary care may help reduce discontinuity in rehabilitation support, consistent with principles of transitional care for older adults (48, 49). However, specific models of caregiver support, follow-up, and cross-level healthcare coordination should be evaluated in future studies before broader implementation.

Participants’ preference for traditional therapies over modern interventions appeared to reflect both accessibility constraints and cultural familiarity. Geographic distance, financial limitations, and familiarity with traditional practices influenced how participants made decisions about pain management and rehabilitation. Rather than indicating simple rejection of modern care, these choices may reflect the interaction between structural barriers and culturally shaped treatment preferences. Participants’ tendency to attribute functional decline to aging and to prioritize younger family members’ needs also shaped rehabilitation decisions. Previous research has shown that perceptions of aging are socially and culturally constructed and may influence how older adults interpret illness and healthcare needs (50, 51). Related work on dignity in later-life care further emphasizes the importance of respecting older adults’ autonomy and personal values when discussing treatment and recovery (52–54). In addition, culturally sensitive communication may help healthcare professionals acknowledge patients’ existing beliefs while clearly explaining the potential benefits and risks of evidence-based rehabilitation approaches (55). Spiritual and folk beliefs were also evident in some participants’ accounts, suggesting that rehabilitation communication should remain respectful of cultural beliefs without assuming that such beliefs should replace formal rehabilitation. Rather than prescribing specific culturally based interventions, future research should examine how culturally responsive rehabilitation support can be integrated with evidence-based care in rural settings.

Taken together, the findings suggest several potential directions for improving home-based rehabilitation support for rural older adults after hip or knee arthroplasty. Rehabilitation information may need to be more accessible to patients with limited health literacy, while post-discharge support could be strengthened through clearer follow-up pathways and more consistent communication between tertiary hospitals and rural primary care providers. Family members may also benefit from basic guidance on how to support rehabilitation safely at home. These implications should be considered as potential directions for future service development rather than established interventions. Their feasibility, effectiveness, and resource requirements should be evaluated in future studies before wider implementation.

This study explores the home-based rehabilitation experiences of rural older adults in China after hip or knee arthroplasty, addressing a gap in the literature on this population. However, several limitations should be noted. First, participants were recruited from one region in Jiangsu Province. The findings may not be fully transferable to rural areas with different cultural beliefs or more severe economic constraints. Second, recruitment was limited to patients who returned to tertiary hospitals for follow-up. This may have excluded those who could not attend because of distance, cost, mobility problems, or lack of caregiver support, and who may have faced the greatest rehabilitation barriers. Third, the cross-sectional design offers only a snapshot of experiences at a particular point in time and does not capture how perceptions, coping strategies, and support needs change over the rehabilitation journey. Fourth, the study included only patient perspectives; interviewing village doctors, family caregivers, and policymakers in future studies could provide a more comprehensive view. Fifth, combining THA and TKA participants may mask procedure-specific differences in precautions, weight-bearing progression, gait training, and exercise prescription. We checked whether the main themes appeared in both groups and noted differences where relevant, but future studies should compare the two groups directly. Sixth, occupational history and occupational risk factors were not collected, although previous physical work and musculoskeletal demands may shape functional limitations, pain, and rehabilitation needs. Future studies should record occupational history, years of physical labor, and specific exposures such as heavy lifting, kneeling, squatting, and prolonged standing. Seventh, socioeconomic status, income, pension or employment status, household financial resources, healthcare expenditure, and rehabilitation affordability were not systematically assessed. Financial hardship was identified only through participants’ accounts, such as treatment, travel, and medicine costs, and their decisions to prioritize family expenses over their own care. Future studies should use structured socioeconomic measures to examine how affordability shapes access, adherence, and coping. Eighth, participants described their experiences retrospectively, which may introduce recall bias, and interviewer influence may have shaped responses. Future studies should address these limitations through broader community-based recruitment, procedure-specific comparisons, and more detailed occupational and socioeconomic assessment, as appropriate.

## 5 Conclusion

Within the context of this single-region qualitative study, the home-based rehabilitation experience of rural older adults after joint replacement is shaped by physical challenges, family dynamics, community resources, health-system gaps, and cultural context. This study not only describes the physical challenges these patients face but also explores their emotional conflicts and cultural adaptation mechanisms. By doing so, it provides preliminary insights for developing context-informed rehabilitation interventions that are both culturally sensitive and practically feasible. Given the geographic limitation to Jiangsu Province, these findings should be transferred to other rural contexts with caution, and further research across diverse regions is warranted. Future interventions may need to consider the emotional and sociocultural dimensions of recovery alongside physical rehabilitation. Context-sensitive strategies that reflect rural patients’ living circumstances and available support may inform future efforts to improve the continuity and accessibility of home-based rehabilitation.

## Data Availability

The raw data supporting the conclusions of this article will be made available by the authors, without undue reservation.
