# Supplementary material for: Home-based rehabilitation after hip or knee arthroplasty among rural older adults in China: a qualitative study
Source: Front Public Health. 2026 Oct 19;14:1957794. doi: 10.3389/fpubh.2026.1957794 (PMC13724545; doi:10.3389/fpubh.2026.1957794)
Supplement: Supplementary file 1 [file Data_Sheet_1.DOCX]

**Supplementary 1 Outline of the semi-structured in-depth interview**

|  | Content |
| --- | --- |
| Section 1 | Background Information  Thank you for agreeing to participate in this study. The purpose of this interview is to understand your experiences and perceptions of home-based rehabilitation after your joint replacement surgery. Your insights will help us identify key challenges and develop effective interventions.  Can you tell me a bit about yourself? How long have you been living in the rural area? |
| Section 2 | Interview Content  1. Can you describe your daily routine during the rehabilitation process? What specific exercises have you been doing as part of your rehabilitation? How do you feel these exercises are helping you?  2. Have you experienced any pain during the rehabilitation process? If yes, can you describe the nature and intensity of the pain? How have you been managing your pain?  3. Have you noticed any changes in your mobility or daily activities since the surgery? What daily tasks have become more difficult for you? How has this affected your overall quality of life?  4. What strategies have you used to cope with the challenges of rehabilitation? Have you sought any support from family, friends, or community members?  5. How has the rehabilitation process affected your mental and emotional well-being? How do you balance your own needs with the needs of your family?  6. What are your hopes and expectations for your recovery in the future? Are there any additional thoughts or experiences you would like to share that we haven't covered? |
| Section 3 | Conclusion  Thank you very much for sharing your experiences and insights with us. Your participation is greatly appreciated and will help us improve the rehabilitation process for others in the future. |
